# Supplementary material for: Toxicity-specific peripheral blood T and B cell dynamics in anti-PD-1 and combined immune checkpoint inhibition
Source: Cancer Immunol Immunother. 2023 Oct 4;72(12):4049–64. doi: 10.1007/s00262-023-03541-0 (PMC10700442; doi:10.1007/s00262-023-03541-0)
Supplement: Supplementary file 1 — Suplementary file information [file 262_2023_3541_MOESM1_ESM.pdf]

Supplementary material

Toxicity-specific peripheral blood T and B cell dynamics in anti-PD-1 and combined immune checkpoint inhibition

Mick J.M. van Eijs,[1,2,#] Rik J. Verheijden,[1] Stefanie A. van der Wees,[2] Stefan Nierkens,[2,3] Anne S.R. van Lindert,[4] Karijn P.M. Suijkerbuijk[1,*], Femke van Wijk;[2,*] on behalf of the UNICIT Consortium[†]

[1] Department of medical oncology, University Medical Center Utrecht, Utrecht, the Netherlands
[2] Center for Translational Immunology, University Medical Center Utrecht, Utrecht, the Netherlands
[3] Princess Máxima Center for Pediatric Oncology, Utrecht, the Netherlands
[4] Department of pulmonology, University Medical Center Utrecht, Utrecht, the Netherlands

* Contributed equally
[#] Corresponding author: Mick J.M. van Eijs. Internal mail no. KC.02.085.2, P.O. Box 85090, 3508 AB, Utrecht, the Netherlands; T: +31 (0)88 757 7183; E: m.j.m.vaneijs-2@umcutrecht.nl

[†] **Collaborators UNICIT consortium**: Linde Meyaard, Jürgen H.E. Kuball, Bas Oldenburg, Jeanette H.W. Leusen.

Supplementary Tables

Supplementary Table 1. Flow cytometry panels

Antigen	Fluorophore	Clone	Company	Catalog number	Lot number(s)	Staining	Dilution
Panel 1 (B cells)							
IgG	FITC	polyclonal	Southern Biotech	2042-02	K2419-Z619E	Surface	500
CD38	PerCP-Cy5.5	HIT2	BD	551400	1159441	Surface	100
CD27	APC	L128	BD	337169	1105983	Surface	50
CD3	AF700	UCHT1	Biolegend	300424	B326013, B279942, B347088	Surface	50
CD19	APC-eF780	HIB19	eBioscience	47-0199-42	2387508	Surface	20
IgM	BV421	G20-127	BD	562618	0328568	Surface	50
Fixable viability dye	eFluor506	-	Fisher Scientific	15560607	2290923	-	1000
CD21	BV711	B-ly4	BD	563163	1146348	Surface	20
CD24	PE-CF594	ML5	BD	562405	1229319	Surface	200
CCR6	PE-Cy7	R6H1	eBioscience	25-1969-42	2304422	Surface	100
Panel 2 (T cell cytotoxicity)							
CD57	FITC	HNK-1	Biolegend	359603	B327318	Surface	100
CD8α	PerCP-Cy5.5	RPA-T8	Biolegend	301032	B334771	Surface	1000
CD3	AF700	UCHT1	Biolegend	300424	B326013, B279942, B347088	Surface	50
CD95	eFluor450	DX2	eBioscience	48-0959-42	2153486	Surface	100
Fixable viability dye	eFluor506	-	Fisher Scientific	15560607	2290923	-	1000
PD-1	BV711	EH12.1	BD	564017	1229849	Surface	100
IgG4 (on-treatment)	biotin	HP6025	Invitrogen	A10663	2309130, 2431369	Surface	50
Streptavidin	BV711	-	Biolegend	405241	B336306	Surface	100
CD4	BV785	RPA-T4	Biolegend	300554	B337916, B344396, B351096	Surface	50
LAG-3	PE	polyclonal	R&D	FAB2319P	AALE1619111	Surface	25
CD45RO	PE-Dazzle	UCHL1	Biolegend	304247	B301149	Surface	400
Granzyme B	APC-Fire750	QA16A02	Biolegend	372210	B337650	Intracellular	50
IFN-γ	PE-Cy7	4S.B3	BD	557844	1117951	Intracellular	200
Panel 3 (T-helper subsets)							
CCR4	FITC	205410	R&D	FAC1567F	LDG0621041	Surface	16.67
CD8α	PerCP-Cy5.5	RPA-T8	Biolegend	301032	B334771	Surface	1000
CD3	AF700	UCHT1	Biolegend	300424	B326013, B279942, B347088	Surface	50
Fixable viability dye	eFluor506	-	Fisher Scientific	15560607	2290923	-	1000
CXCR3	BV605	G025H7	Biolegend	353728	B329131, B339512, B349628	Surface	12.5
CD45RA	BV711	HI100	Biolegend	304138	B334528	Surface	500
CD4	BV785	RPA-T4	Biolegend	300554	B337916, B344396, B351096	Surface	50
CCR5	PE	eBioT21/8	eBioscience	12-1957-42	2172554	Surface	50
CD45RO	PE-Dazzle	UCHL1	Biolegend	304247	B301149	Surface	400
Ki67	AF647	B56	BD	558615	0342573, 1225152	Intracellular	50
FOXP3	eFLuor450	PCH101	eBioscience	48-4776-42	2299833	Intracellular	50
Panel 4 (naive/memory subsets 1)							
CD95	FITC	DX2	BD	340479	1067724	Surface	
CD8α	PerCP-Cy5.5	RPA-T8	Biolegend	301032	B334771	Surface	1000
CD3	AF700	UCHT1	Biolegend	300424	B326013, B279942, B347088	Surface	50
CD27	APC-eF780	O323	eBioscience	47-0279-42	2241980, 2452256	Surface	20
Fixable viability dye	eFluor506	-	Fisher Scientific	15560607	2290923	-	1000
CD31	BV605	WM59	BD	562855	1099569, B283706	Surface	20
CD45RA	BV711	HI100	Biolegend	304138	B334528	Surface	500
CD4	BV785	RPA-T4	Biolegend	300554	B337916, B344396, B351096	Surface	50
Ki67	AF647	B56	BD	558615	0342573,1225152	Intracellular	50
IL-2	PB	MQ1-17H12	Biolegend	500324	B317866	Intracellular	100
IL-17A	PE	eBio64DEC17	eBioscience	12-7179-42	2331144	Intracellular	50
IL-8	PE-CF594	G265-8	BD	563531	0311622	Intracellular	200
Panel 5 (regulation)							
ICOS	FITC	C398.4A	Biolegend	313506	B311819	Surface	400
TIGIT	PerCP-Cy5.5	MBSA43	eBioscience	46-9500-42	2284173	Surface	50
CD3	AF700	UCHT1	Biolegend	300424	B326013, B279942, B347088	Surface	50
CD45RA	APC-Cy7	HI100	Sony Biotech	2120640	235800	Surface	50
Fixable viability dye	eFluor506	-	Fisher Scientific	15560607	2290923	-	1000
CD4	BV785	RPA-T4	Biolegend	300554	B337916, B344396, B351096	Surface	50
CCR8	PE	L263G8	Biolegend	360603	B334779	Surface	100
CD25	PE-Cy7	M-A251	BD	557741	1068706	Surface	25
CTLA-4	APC	BNI3	BD	555855	0335549	Intracellular	12.5
FOXP3	eFLuor450	PCH101	eBioscience	48-4776-42	2299833	Intracellular	50
T-bet	PE-CF594	O4-46	BD	562467	1209448	Intracellular	50
Panel 6 (T cell-monocyte ratio)							
CD3	PerCP-Cy5.5	UCHT1	Biolegend	300430	B331881	Surface	100
CD14	APC-eF780	61D3	eBioscience	47-0149-42	2284099	Surface	40
Panel 7 (naive/memory subsets 2)							
CD8	FITC	RPA-T8	BD	555366	1109087	Surface	100
CD3	AF700	UCHT1	Biolegend	118300424	B326013, B279942, B347088	Surface	50
CCR7	APC-Fire750	G043H7	Biolegend	353246	B338294	Surface	12.5
Fixable viability dye	eFluor506	-	Fisher Scientific	15560607	2290923	-	1000
CD4	BV785	RPA-T4	Biolegend	300554	B337916, B344396, B351096	Surface	50
CD45RO	PE-Dazzle	UCHL1	Biolegend	304247	B301149	Surface	200

Supplementary Table 2. Luminex analytes

Target	Full protein name
IL-5	Interleukin-5
IL-6	Interleukin-6
IL-7	Interleukin-7
IL-10	Interleukin-10
IL-12	Interleukin-12
IL-13	Interleukin-13
IL-17	Interleukin-17
IL-21	Interleukin-21
IL-23	Interleukin-23
IL-33	Interleukin-33
TNF-α	Tumor necrosis factor alpha
IFN-γ	Interferon gamma
APRIL	A proliferation-inducing ligand
CCL2 (MCP1)	C-C motif ligand 2 (monocyte chemoattractant protein 1)
CCL4 (MIP-1β)	C-C motif ligand 4 (macrophage inflammatory protein)
CCL17 (TARC)	C-C motif ligand 17 (thymus- and activation-regulated chemokine)
CXCL9	C-X-C motif ligand 9
CXCL10	C-X-C motif ligand 10
CXCL13	C-X-C motif ligand 13
sILR2	Soluble interleukin-2 receptor
GzmB	Granzyme B
TGF-β1 (LAP)	Transforming growth factor beta 1 (latency-associated peptide)
TACI	Transmembrane activator and CAML interactor

Supplementary Table 3. Extended characteristics of patients and healthy donors

	**Anti-PD-1 NOTx (N=13)**	**Anti-PD-1 TOX (N=11)**	**cICI NOTx (N=10)**	**cICI TOX (N=10)**	**Healthy donor (N=10)**	***P* value**
Timepoint 2 available, N (%)[a]	13 (100.0)	7 (63.6)	10 (100.0)	6 (60.0)	N/A	**0.011**
Time-to-timepoint 2 (weeks)						
Median (IQR)	3.0 (3.0-4.0)	4.0 (3.5-4.0)	3.0 (3.0-3.0)	3.0 (3.0-3.0)	N/A	**<0.01**
Time-to-timepoint 3 (weeks)						
Median (IQR)	6.0 (6.0-8.0)	N/A	6.0 (6.0-6.0)	N/A	N/A	0.11
Best overall tumor response per RECIST1.1						
Not evaluable	2 (15.4)	3 (27.3)	0 (0.0)	2 (20.0)	N/A	0.51
Progressive disease	6 (46.2)	1 (9.1)	5 (50.0)	3 (30.0)		
Stable disease	2 (15.4)	4 (36.4)	1 (10.0)	2 (20.0)		
Partial response	2 (15.4)	2 (18.2)	4 (40.0)	2 (20.0)		
Complete response	1 (7.7)	1 (9.1)	0 (0.0)	1 (10.0)		
Baseline ECOG performance status						
0	7 (53.8)	4 (36.4)	4 (40.0)	5 (50.0)	N/A	0.86
1	5 (38.5)	5 (45.5)	4 (40.0)	5 (50.0)		
2	1 (7.7)	2 (18.2)	2 (20.0)	0 (0.0)		
irAE types[b]						
Arthritis	N/A	2 (18.2)	N/A	0 (0.0)	N/A	**0.010**
Colitis		0 (0.0)		3 (30.0)		
Dermatitis		0 (0.0)		2 (20.0)		
Duodenitis		1 (9.1)		1 (10.0)		
Gastritis		0 (0.0)		1 (10.0)		
Hepatitis		1 (9.1)		4 (40.0)		
Hypophysitis		0 (0.0)		2 (20.0)		
Meningitis		0 (0.0)		3 (30.0)		
Myocarditis		1 (9.1)		0 (0.0)		
Myositis		2 (18.2)		0 (0.0)		
Nephritis		1 (9.1)		1 (10.0)		
Pancreatitis		1 (9.1)		1 (10.0)		
Pneumonitis		2 (18.2)		0 (0.0)		
Thyroiditis		1 (9.1)		1 (10.0)		

Groups are compared by Kruskal-Wallis (continuous data) or Fisher's exact test (categorical data). Abbreviations: anti-PD-1 denotes 'anti-PD-1 monotherapy', cICI 'combined immune checkpoint inhibition', CTCAE 'Common Terminology Criteria for Adverse Events', irAE 'immune-related adverse event'; N/A 'not applicable', NOTx 'no clinically relevant irAEs', TOX 'with clinically relevant irAEs', yr 'years'. [a] In 2/4 cICI TOX patients and 3/4 anti-PD-1 TOX patients Timepoint 2 sample is missing because irAE(s) developed after one cycle of ICI .[b]Numbers exceed 100% because patients may have more than one irAE simultaneously.

Supplementary Table 4. Top 10 parameters contributing to principal component 1 and 2

Top 10 unique read-out parameters	Principal component (PC) 1	Principal component (PC) 2
1 (highest loading score)	CD45RO$^-$ CCR7$^+$ naïve of CD8$^+$ T cells	Ki67$^+$ of CD4$^+$ T cells
2	granB$^+$ of PD-1$^-$LAG-3$^-$ CD95$^+$ CD8$^+$ T cells	Ki67$^+$ of CD8$^+$ T cells
3	granB$^+$ of CD57$^-$ CD8$^+$ T cells	Ki67$^+$ of CD45RA$^-$**CD27$^-$ effector** memory CD4$^+$ T cells
4	granB$^+$ of PD-1$^+$LAG-3$^-$ CD95$^+$ CD8$^+$ T cells	Ki67$^+$ of CXCR3$^+$CCR4$^-$ Th1-associated CD45RO$^+$ memory CD4$^+$ T cells
5	CD57$^+$ of CD8$^+$ T cells	Ki67$^+$ of CXCR3$^+$ CD45RO$^+$ memory CD8$^+$ T
6	CD45RA$^+$CD27$^-$ T_{EMRA} of CD8$^+$ T cells	Ki67$^+$ of CD45RA$^-$**CD27$^-$ effector** memory CD8$^+$ T cells
7	CD4$^+$ T cells of CD3$^+$ T cells	Ki67$^+$ of CD45RA$^+$CD27$^-$ CD8$^+$ T_{EMRA} cells
8	CD8$^+$ T cells of CD3$^+$ T cells	Ki67$^+$ of CD45RA$^-$**CD27$^+$ central/effector** memory CD8$^+$ T cells
9	IFN-γ^+ of CD4$^+$ T cells	Ki67$^+$ of CD45RA$^-$**CD27$^+$ central/effector** memory CD4$^+$ T cells
10 (lowest loading score)	CD57$^+$ of CD95$^+$ CD8$^+$ T cells	Ki67$^+$ of CXCR3$^-$CCR4$^+$ Th2-associated CD4$^+$ T cells

All parameters represent parental percentages based on manual gating. Each biologically unique population is included only once in the top 10. For example, the percentage CD4$^+$ of CD3$^+$ T cells was assessed in multiple panels and each panel-specific parameter contributed individually to the top 10, but percentage CD4$^+$ of CD3$^+$ T cells is included only once in PC1 top 10. Abbreviations: EMRA denotes 'effector memory re-expressing CD45RA', granB 'granzyme B'.

Supplementary Figures

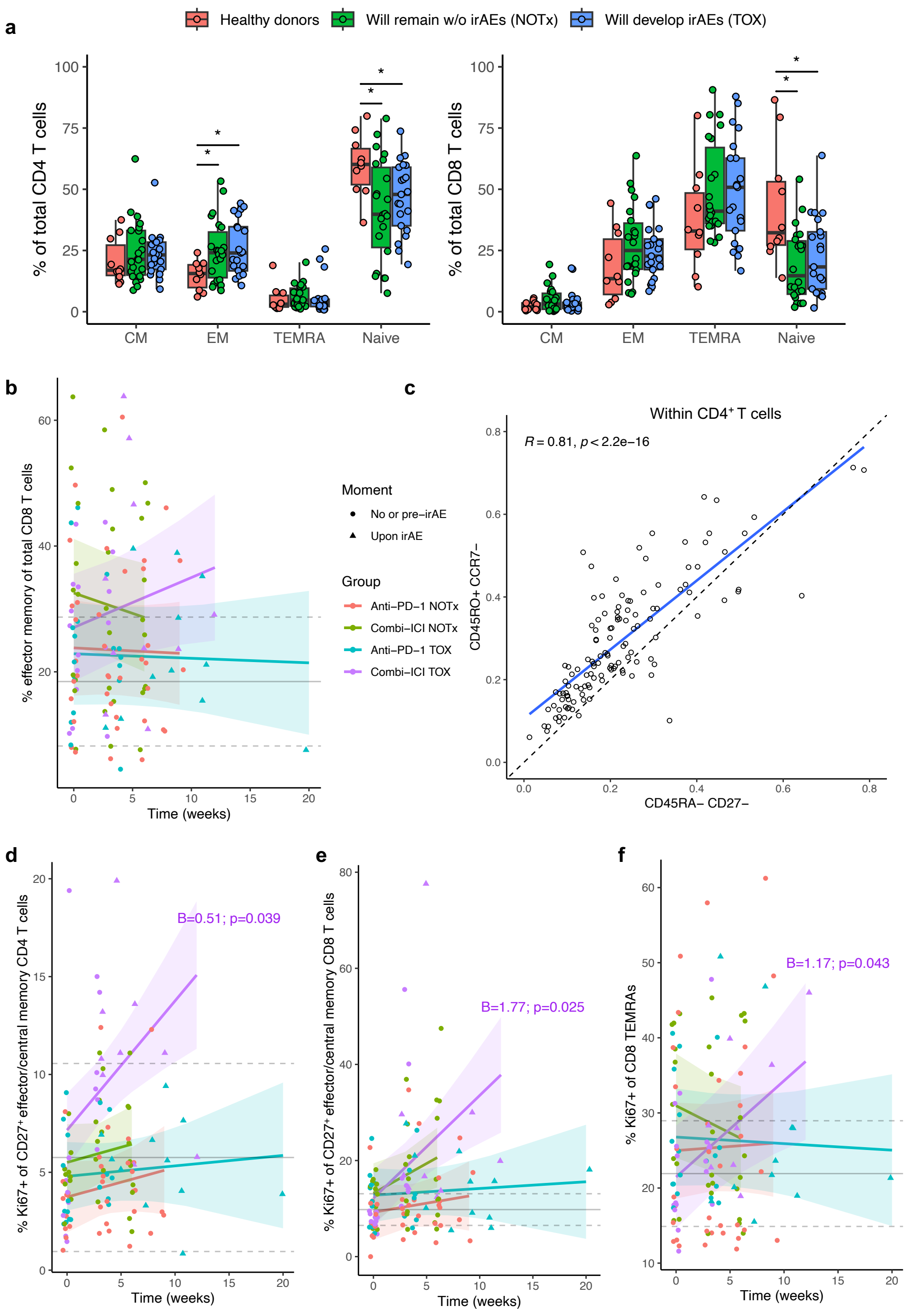

Supplementary Figure 1 Patients with cancer have different baseline naïve/memory subset distribution than healthy donors, while over time all CD4$^+$ and CD8$^+$ memory subsets in combined-ICI treated patients with toxicity demonstrate enhanced proliferation compared to other patient groups. (**a**) Baseline abundance of central memory (CM), effector memory (EM), TEMRA and naive subsets in CD4$^+$ T cells (left) and CD8$^+$ T cells (right). Comparisons by Kruskal-Wallis tests and Dunn's post-hoc tests with Benjamini-Hochberg correction for multiple testing. (**b**) Percentage CD8$_{EM}$ of total CD8$^+$ T cells over time. Only significant coefficients for the interaction term with time ('B'), indicating statistically significant change compared to other groups, from mixed-effects models are shown. Gray solid and dashed lines indicate mean healthy donor level with 95% confidence interval. (**c**) Correlation between fraction CD45RO$^+$CCR7$^-$ CD4$_{EM}$ and fraction CD45RA$^-$CD27$^-$ CD4$^+$ T cells of all CD4$^+$ T cells across all samples and timepoints (by Pearson correlation); the dashed line represents the diagonal (y=x). (**d-f**) Percentage proliferating of total CD27$^+$ CD4$_{EM/CM}$ (**d**), CD27$^+$ CD8$_{EM/CM}$ (**e**) and CD8$_{EMRA}$ (**f**) T cells over time. irAE: immune-related adverse event, TOX: with irAEs, NOTx: without irAEs, *P<0.05.

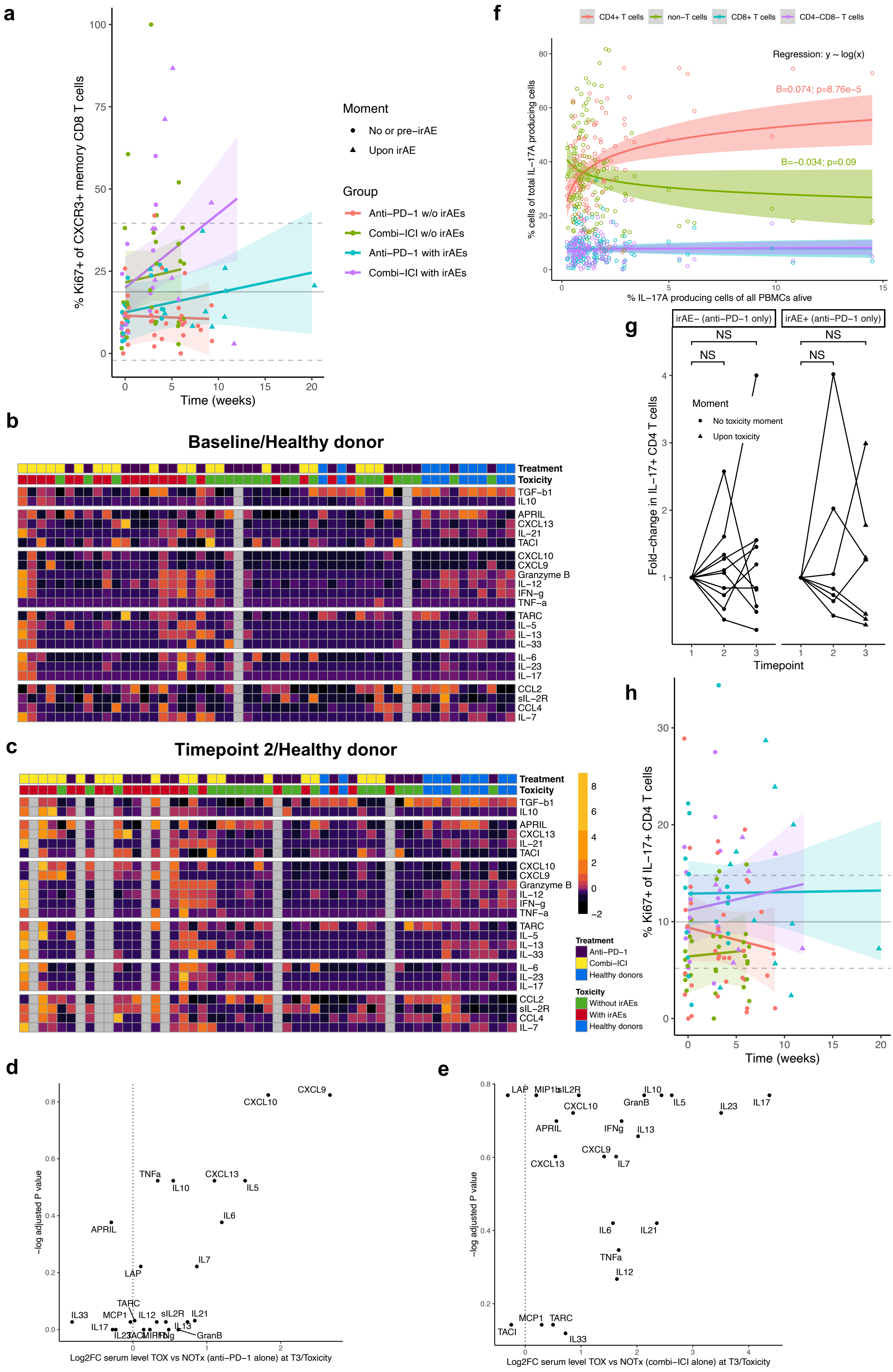
a
% Ki67+ of CXCR3+ memory CD8 T cells
Time (weeks)
Moment
No or pre−irAE
Upon irAE
Group
Anti−PD−1 w/o irAEs
Combi−ICI w/o irAEs
Anti−PD−1 with irAEs
Combi−ICI with irAEs
b
Baseline/Healthy donor
Treatment
Toxicity
TGF−b1
IL10
APRIL
CXCL13
IL−21
TACI
CXCL10
CXCL9
Granzyme B
IL−12
IFN−g
TNF−a
TARC
IL−5
IL−13
IL−33
IL−6
IL−23
IL−17
CCL2
sIL−2R
CCL4
IL−7
c
Timepoint 2/Healthy donor
Treatment
Anti−PD−1
Combi−ICI
Healthy donors
Toxicity
Without irAEs
With irAEs
Healthy donors
d
−log adjusted P value
Log2FC serum level TOX vs NOTx (anti−PD−1 alone) at T3/Toxicity
CXCL9
CXCL10
TNFa
IL10
CXCL13
IL5
IL6
APRIL
IL7
LAP
TARC
IL33
MCP1
IL12
sIL2R
IL21
IL17
IL13
GranB
e
−log adjusted P value
Log2FC serum level TOX vs NOTx (combi−ICI alone) at T3/Toxicity
LAP
MIP1b
sIL2R
IL10
IL5
IL23
IL17
GranB
CXCL10
APRIL
IFNg
IL13
CXCL9
CXCL13
IL7
IL6
IL21
TNFa
IL12
MCP1
TARC
TACI
IL33
f
CD4+ T cells
non−T cells
CD8+ T cells
CD4−CD8− T cells
Regression: y ~ log(x)
B=0.074; p=8.76e−5
B=−0.034; p=0.09
% cells of total IL−17A producing cells
% IL−17A producing cells of all PBMCs alive
g
irAE− (anti−PD−1 only)
irAE+ (anti−PD−1 only)
NS
Moment
No toxicity moment
Upon toxicity
Fold−change in IL−17+ CD4 T cells
Timepoint
h
% Ki67+ of IL−17+ CD4 T cells
Time (weeks)

Supplementary Figure 2 Especially Th1- and Th17-associated cytokines increase towards toxicity after combined ICI, while for anti-PD-1 associated toxicity this is limited to increase in Th1-associated cytokines without changes in IL-17$^+$ producing cells compared to patients without toxicity. (**a**) Percentage proliferating of CXCR3$^+$ CD8$^+$ T cells over time. Mixed-effects models showed no significant interactions with time. Gray solid and dashed lines indicate mean healthy donor level with 95% confidence interval. (**b**,**c**) Heatmaps showing serum levels of cytokines and chemokines at baseline (**b**) and Timepoint 2 (**c**) or in healthy donors. Data are scaled by individual proteins across timepoints (see **Fig. 3c**), enabling direct comparison of individual proteins between three timepoints. Grey columns indicate missing timepoints; column order (patients) is as in **Fig. 3c**. (**d,e**) Volcano plots showing relative increase in serum protein levels at Timepoint 3/Toxicity in patients with irAEs (TOX) versus patients without irAEs (NOTx) for (**d**) anti-PD-1 monotherapy and (**e**) combined ICI treated patients. Full protein names are in **Supplementary Table 2**. (**f**) Plot showing relation between increasing IL-17A-producing cells (as percentage of all PBMCs) and relative contribution of CD4$^+$ T cells (Th17 cells) to total IL-17 production by PBMCs, compared to CD3$^-$ non-T cells (including monocytes), CD8$^+$ T cells and CD4$^-$CD8$^-$ T cells. (**g**) Fold-change in percentage IL-17$^+$ of CD4$^+$ T cells relative to baseline in anti-PD-1-treated patients without irAEs (left; NOTx) and with (right; TOX) irAEs. (**h**) Percentage proliferating of IL-17$^+$ CD4$^+$ T cells over time. The same legend, graphical approach and statistics as in (**a**) apply; no significant interactions with time. irAE: immune-related adverse event, NS: not significant, TOX: with irAEs, NOTx: without irAEs.

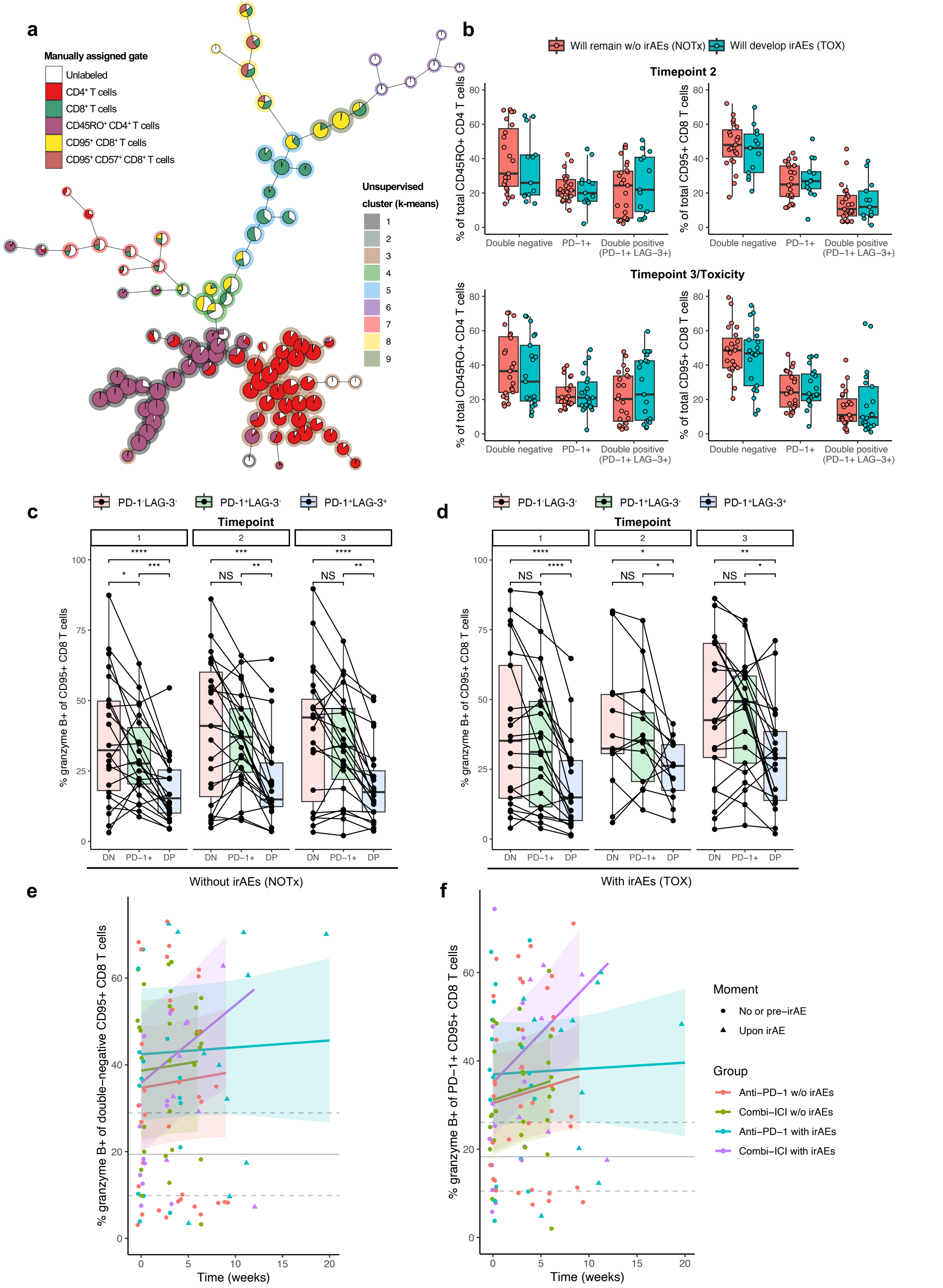

Supplementary Figure 3 Relative abundance of PD-1^{+}LAG-3^{+} double positive (DP) CD8^{+} memory T cells while on-treatment remains unchanged, but towards toxicity especially these DP CD8^{+} memory T cells show increased cytotoxic potential. (**a**) FlowSOM visualization created with concatenated file containing 10% of CD3^{+} T cells from all patients at Timepoint 3/Toxicity and healthy donors, overlaid with k-means clustering results and manually assigned gates. (**b**) Percentages of CD45RO^{+} CD4^{+} (left) and CD95^{+} CD8^{+} (right) T cells positive for PD-1 alone or PD-1 and LAG-3 at Timepoint 2 (top) or Timepoint 3/Toxicity (bottom). (**c**,**d**) Percentages of CD95^{+}CD8^{+} T cells producing granzyme B over time after PMA/ionomycin stimulation,

depending on inhibitory receptor expression pattern, in patients without (NOTx; **c**) or with irAEs (TOX; **d**); anti-PD-1- and cICI-treated patients are combined. Comparisons by pairwise paired Wilcoxon tests with Benjamini-Hochberg correction for multiple testing. (**e**,**f**) Percentage of granzyme B producing (**e**) PD-1$^-$LAG-3$^-$ double-negative and (**f**) PD-1$^+$ CD95$^+$CD8$^+$ T cells over time analyzed by mixed models; no significant interactions with time. Gray solid and dashed lines indicate mean healthy donor level with 95% confidence interval. irAE: immune-related adverse event, NS: not significant, TOX: with irAEs, NOTx: without irAEs. *P<0.05, **P<0.01, ***P<0.001, ****P<0.0001.

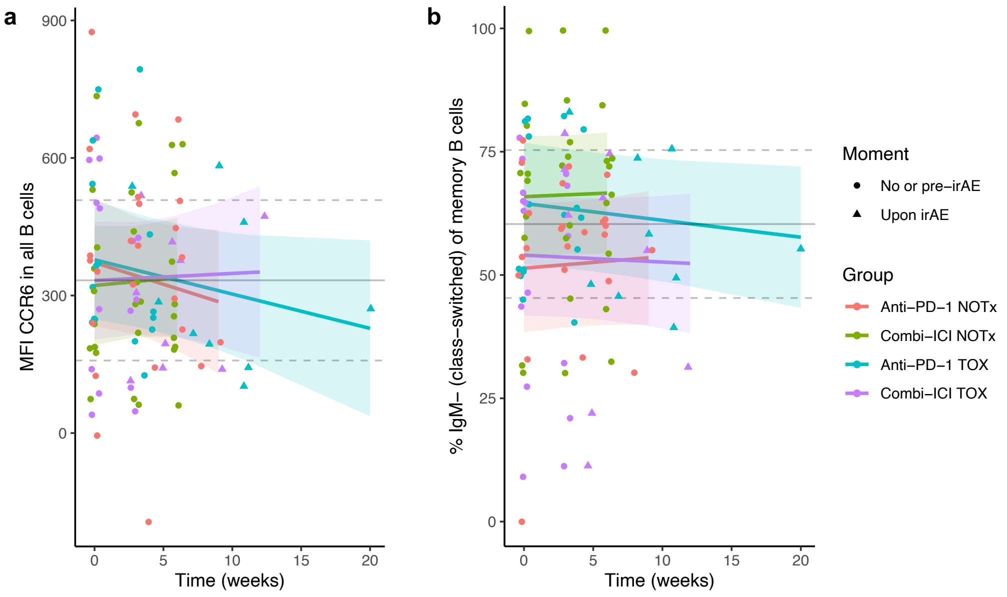

Supplementary Figure 4 Extent of B cell activation and class-switching do not change among groups over time. (**a**) Median fluorescence intensity (MFI) of CCR6 in all B cells over time and (**b**) Percentage IgM$^-$ class-switched of CD27$^+$ memory B cells over time. Mixed-effects models showed no significant interactions with time. Gray solid and dashed lines indicate mean healthy donor level with 95% confidence interval. irAE: immune-related adverse event, TOX: with irAEs, NOTx: without irAEs.

a

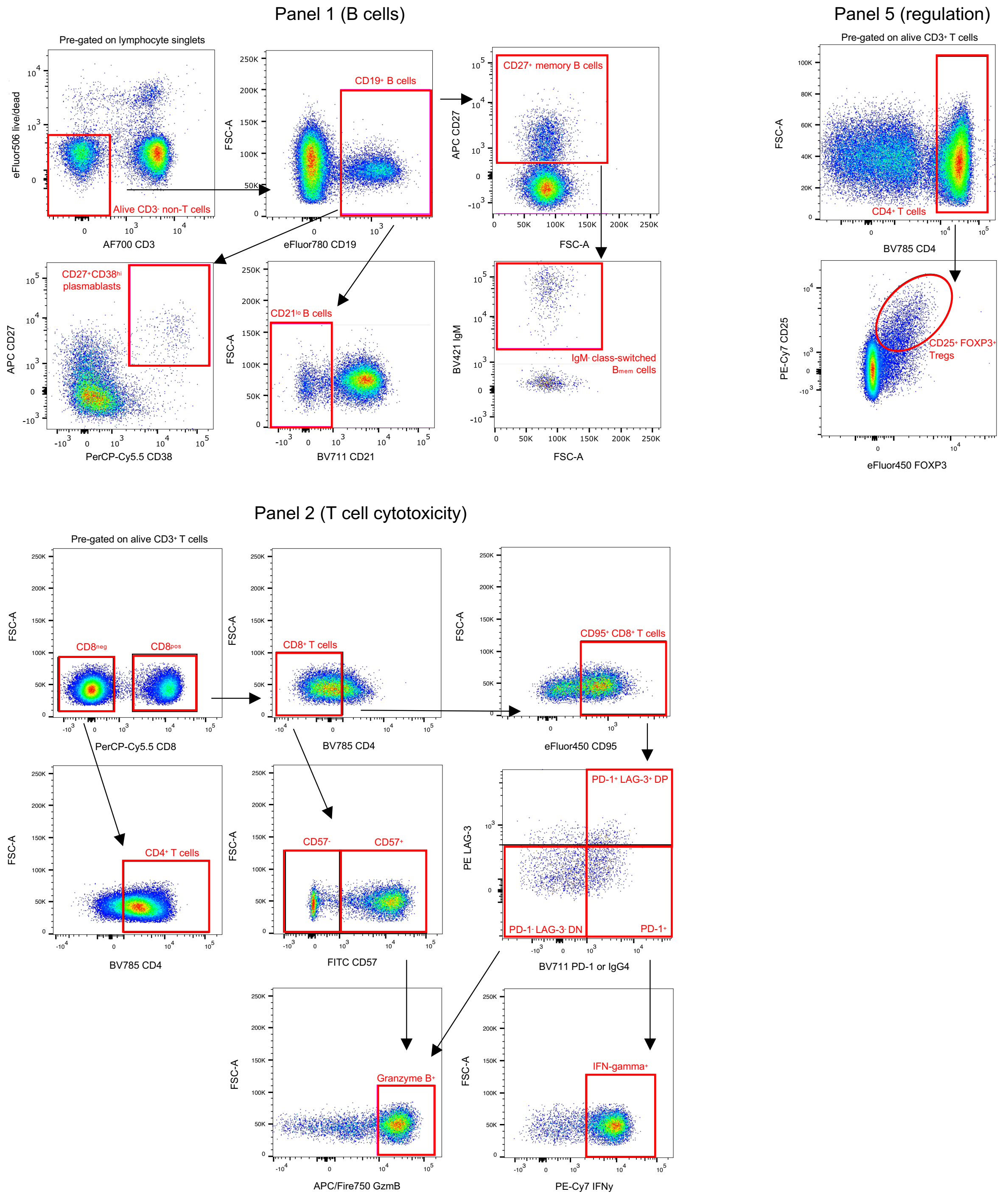
Panel 1 (B cells)
Pre-gated on lymphocyte singlets
eFluor506 live/dead
AF700 CD3
Alive CD3- non-T cells
FSC-A
eFluor780 CD19
CD19+ B cells
APC CD27
CD27+ memory B cells
FSC-A
CD27+CD38hi plasmablasts
PerCP-Cy5.5 CD38
CD21lo B cells
BV711 CD21
BV421 IgM
IgM- class-switched Bmem cells
Panel 5 (regulation)
Pre-gated on alive CD3+ T cells
CD4+ T cells
BV785 CD4
PE-Cy7 CD25
CD25+ FOXP3+ Tregs
eFluor450 FOXP3
Panel 2 (T cell cytotoxicity)
Pre-gated on alive CD3+ T cells
CD8neg
CD8pos
PerCP-Cy5.5 CD8
CD8+ T cells
BV785 CD4
CD95+ CD8+ T cells
eFluor450 CD95
CD4+ T cells
BV785 CD4
CD57-
CD57+
FITC CD57
PE LAG-3
PD-1+ LAG-3+ DP
PD-1- LAG-3- DN
PD-1+
BV711 PD-1 or IgG4
Granzyme B+
APC/Fire750 GzmB
IFN-gamma+
PE-Cy7 IFNy

b

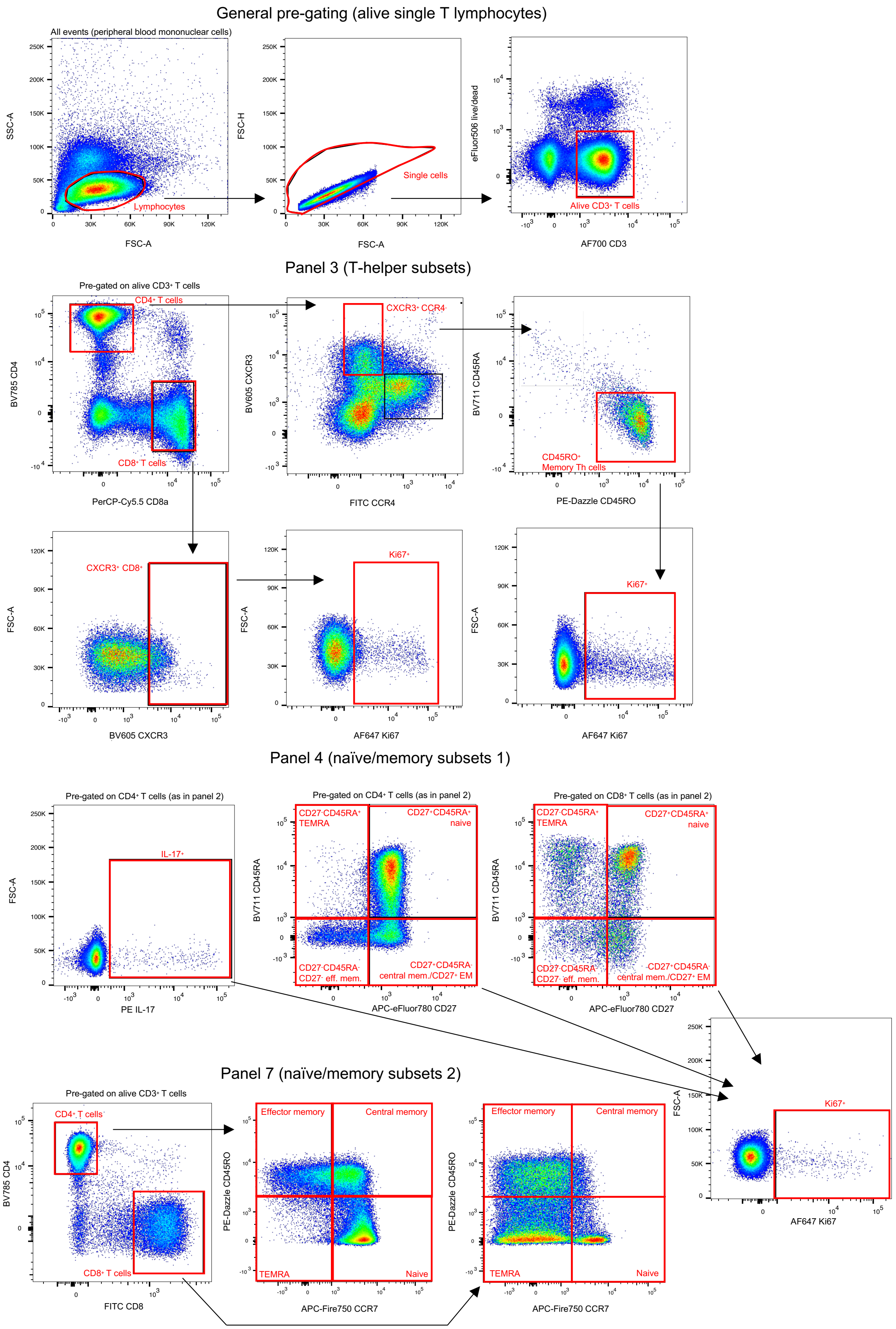
General pre-gating (alive single T lymphocytes)
All events (peripheral blood mononuclear cells)
SSC-A
FSC-A
Lymphocytes
FSC-H
Single cells
eFluor506 live/dead
AF700 CD3
Alive CD3+ T cells
Panel 3 (T-helper subsets)
Pre-gated on alive CD3+ T cells
BV785 CD4
PerCP-Cy5.5 CD8a
CD4+ T cells
CD8+ T cells
BV605 CXCR3
FITC CCR4
CXCR3+ CCR4-
BV711 CD45RA
PE-Dazzle CD45RO
CD45RO+
Memory Th cells
FSC-A
BV605 CXCR3
CXCR3+ CD8+
AF647 Ki67
Ki67+
Panel 4 (naïve/memory subsets 1)
Pre-gated on CD4+ T cells (as in panel 2)
PE IL-17
IL-17+
APC-eFluor780 CD27
CD27- CD45RA+
TEMRA
CD27+ CD45RA+
naive
CD27- CD45RA-
CD27- eff. mem.
CD27+ CD45RA-
central mem./CD27+ EM
Pre-gated on CD8+ T cells (as in panel 2)
Panel 7 (naïve/memory subsets 2)
Pre-gated on alive CD3+ T cells
FITC CD8
PE-Dazzle CD45RO
APC-Fire750 CCR7
Effector memory
Central memory
TEMRA
Naive

Supplementary Figure 5 Representative flow cytometry plots illustrating gating strategies. (**a**) Panel 1 (B cells), Panel 2 (T cell cytotoxicity) and Panel 5 (regulation). Pre-gating strategy for Panels 2 and 5 is in (**b**). (**b**) General pre-gating (alive single T lymphocytes), Panel 3 (T-helper subsets), Panel 4 (naïve/memory subsets 1) and Panel 7 (naïve/memory subsets 2).
